# Roots of *Erigeron annuus* Attenuate Acute Inflammation as Mediated with the Inhibition of NF-**κ**B-Associated Nitric Oxide and Prostaglandin E_2_ production

**DOI:** 10.1155/2013/297427

**Published:** 2013-02-24

**Authors:** Mi Jeong Jo, Jong Rok Lee, Il Je Cho, Young Woo Kim, Sang Chan Kim

**Affiliations:** ^1^Medical Research Center for Globalization of Herbal Formulation, College of Oriental Medicine, Daegu Haany University, Daegu 706-828, Republic of Korea; ^2^Department of Herbal Pharmaceutical Engineering, Daegu Haany University, Kyung-San, Kyung-Buk 712-715, Republic of Korea

## Abstract

*Erigeron annuus* is a naturalized plant belonging to Compositae (asteraceae) family, which is called the annual fleabane, and commonly found at meadows and roadside. This study investigated the anti-inflammatory effects of the extract of *E. annuus* roots (EER), as assessed by the paw edema formation and histological analysis in rat, and the productions of nitric oxide (NO), prostaglandin E_2_ (PGE_2_), and pro-inflammatory cytokines in Raw264.7 murine macrophages. Carrageenan treatment promoted infiltration of inflammatory cells and caused swelling in the hind paw. Oral administrations of EER (0.3 g/kg and 1 g/kg) attenuated acute inflammation similar to the result using dexamethasone (1 mg/kg). Treatment of macrophages with lipopolysaccharide (LPS) simulated inflammatory condition: LPS significantly increased the productions of NO, PGE_2_, and proinflammatory cytokines. EER suppressed activation of macrophages, preventing the induction of iNOS and COX-2 protein expressions. LPS treatment induced phosphorylation of I-**κ**B**α** and increased the level of nuclear NF-**κ**B protein, both of which were suppressed by concomitant treatment of EER. In conclusion, EER ameliorated acute inflammation in rats, and the induction of NO, PGE_2_, and proinflammatory cytokines in Raw264.7 cells. EER's effects may be associated with its inhibition of NF-**κ**B activation, suggesting its effect on inflammatory diseases.

## 1. Introduction

Inflammation of human body is one of the most important biological responses to harmful stimuli (e.g., pathogens or damaged cells) during the host defense mechanism. Acute inflammatory response initiates pathological process of numerous disorders such as infection, bacterial sepsis, cancer, and chronic inflammation in specific organ (i.e., hepatitis and arthritis) [1–6]. In the progression of inflammation, the representative pathological symptoms are characterized by an increased blood flow to the peripheral tissue by augmentation of vascular permeability, which leads to cellular infiltration and swelling [7]. 

Macrophage is one of the most important phagocytic cells and plays a crucial role in the process of inflammation by producing proinflammatory mediators. Lipopolysaccharide (LPS) is the major component of outer membrane of gram-negative bacteria and has the ability to directly activate macrophages to secrete the inflammatory mediators such as NO and PGE_2_ [8–12]. Nitric oxide (NO) is produced by three distinct isoforms of nitric oxide synthases (NOS) (i.e., neural (nNOS), endothelial (eNOS), and inducible (iNOS)). Among them, iNOS is a key regulator for inducing a large quantity of NO during inflammation and is indicated in many cell types such as macrophage, endothelial, and epithelial cells [13, 14]. PGE_2_ is also another important inflammatory mediator which is produced from arachidonic acid through the catalytic reaction by COX-2 [15, 16]. It has been shown that iNOS and COX-2 are transcriptionally activated by NF-*κ*B, one of the most important transcription factors regulating the immune responses, cell adhesion, and survival [17–19].

*Erigeron annuus* (*E. annuus*) is a naturalized plant belonging to Compositae (Asteraceae) family and commonly found at meadows and roadside. In traditional oriental medicine, *E. annuus* has been used to treat indigestion, epidemic hepatitis, enteritis, lymphadenitis, and hematuria [20, 21]. However, the effects of *E. annuus *roots have not been elucidated. In this study, we investigated the potential effect of the extract of *E. annuus *roots (EERs) on acute inflammation induced by carrageenan injection in rats. Furthermore, this study identified EER as an active component having an ability of the inhibitory effects on LPS-stimulated iNOS and COX-2 expressions as well as TNF-*α* and IL-1*β* productions in RAW264.7 macrophages. In view of importance of the inflammation in the process of human disease, these results show the pharmacological effects of EER in terms of the treatment of inflammatory diseases. 

## 2. Materials and Methods

### 2.1. Preparation of the EER

*E. annuus* was collected by ourselves in May 2011 from Sangdong, Suseonggu, Daegu, Korea, and identified by Professor Sang Chan, Kim Ph.D. (College of Oriental Medicine, Daegu Haany University, Korea). The roots part of *E. annuus* were washed twice with water and then dried in the air. EERs were prepared with methanol at room temperature for 72 h. The extract was filtered through a 0.2 *μ*m filter (Nalgene, New York, NY, USA), lyophilized, and stored at −20°C until use. The amount of EERs was estimated by the dried weight of lyophilized EER. The yield of lyophilized EER was 0.89%. 

### 2.2. Analysis of EER

Analysis of EER was performed on an Agilent 6890 gas chromatography equipped with a 5975 GC/MS selective detector (Agilent, CA, USA). Separations were performed in a 30 m length × 0.25 mm i.d. and 0.25 *μ*m film thickness fused silica capillary column HP-5MS (Agilent, CA, USA). The carrier gas was ultrapure helium with a flow of 1 mL/min, and the splitless injector temperature was set as 280°C. The column temperature program was the initial temperature of 70°C for 4 min and increased by 2°C/min 70 to 100°C (held 2 min). After that the temperature was varied from 100 to 200°C at 5°C/min (held 20 min) and increased to 280°C (held 5 min) at 10°C/min, in a total run time of 73 min. Mass spectral analyses were performed using the NIST05 library resident in the computer. Percentage composition was calculated using the area normalization method (see Figure S1 in Supplementary Material available online at http://dx.doi.org/10.1155/2013/297427).

### 2.3. Materials

Anti-NF-*κ*B p65 and horseradish peroxidase-conjugated goat antirabbit were supplied from Santa Cruz Biotechnology (Santa Cruz, CA, USA). Antiphospho-I-*κ*B*α* (p-I-*κ*B*α*), anti-Lamin A, horseradish peroxidase-conjugated antimouse, and antigoat, IgGs were purchased from Cell Signaling (Beverely, MA, USA). Antimurine iNOS was purchased from BD Bioscience (San Jose, CA, USA). Anti-COX-2 antibody was purchased from Cayman (Ann Arbor, Mi, USA). Polyethylene glycol number 400 (PEG) solution, carrageenan, dexamethasone, and other reagents were purchased from Sigma Chemical Co. (St. Louis, MO, USA).

### 2.4. Animal Experiment

Animal experiments were conducted under the guidelines of the Institutional Animal Care and Use Committee (IACUC) at Daegu Haany University [12]. Sprague-Dawley rats at 4 weeks of age (male, 80–100 g) were provided from Hyochang Sience (Daegu, Korea), acclimatized for 1 week, and maintained in a clean room at the Animal Center for Pharmaceutical Research, College of Oriental Medicine, Daegu Haany University. Animals were caged under the supply of filtered pathogen-free air, commercial rat chow (Purina, Korea), and water ad libitum at a temperature between 20 and 23°C with 12 h light and dark cycles and relative humidity of 50%.

### 2.5. Carrageenan-Induced Paw Edema

Rats (*N* = 20) were randomly divided into four groups, and thus each group consisted of five animals. EER, dissolved in 40% polyethylene glycol (PEG) number 400, was orally administered to rats at the dose of 0.3 or 1 g/kg/day for four consecutive days. Dexamethasone (1 mg/kg/day), an anti-inflammatory drug, was used as a positive control [22]. To induce acute phase inflammation in paw, rats were injected subcutaneously into the right hind paw with a 1% solution of carrageenan dissolved in saline 30 min after last treatment of vehicle or EER. The paw volumes were measured up to 4 h after the injection at intervals of 1 h. The hind paw volume was determined volumetrically by measuring with a plethysmometer (Ugo Basile, VA, Italy). After euthanasia using ether, the hind paw samples were collected.

### 2.6. Histological Process

The hind paw skins *ventrum pedis* skins were separated and fixed in 10% neutral buffered formalin, then embedded in paraffin, sectioned (3~4 *μ*m), and stained with hematoxylin and eosin (H and E) [23]. The histopathological profiles of each sample were observed under light microscope (Nikon, Japan).

### 2.7. Histomorphometry

The thicknesses of *ventrum pedis* skins (from epidermis to dermis, keratin layers were excluded) were measured using automated image analyzer (DMI-300 Image Processing, DMI, Korea) under magnification 40 of microscopy (Nikon, Japan) at prepared skin histological samples as mm/paw. The infiltrated inflammatory cells were also counted using automated image analyzer as cells/mm^2^ of dermis under magnification 200 of microscopy [23].

### 2.8. Cell Culture

Raw264.7 cell, a murine macrophage cell line, was obtained from American Type Culture Collection (Rockville, MD, USA). The cells were maintained in Dulbecco's Modified Eagle's Medium (DMEM) containing 10% fetal bovine serum (FBS), 50 U/mL penicillin, and 50 mg/mL streptomycin at 37°C in a humidified atmosphere with 5%  CO_2_. For all experiments, the cells were grown to 80–90% confluency and were subjected to no more than 20 cell passages. Raw264.7 cells were incubated with 1 *μ*g/mL LPS (*Escherichia coli* 026:B6, Sigma, St. Louis, MO, USA). The cells were incubated in the medium without 10% FBS for 24 h and then exposed to LPS or LPS + EER for the indicated time periods. EER dissolved in dimethyl sulfoxide was added to the incubation medium 1 h prior to the addition of LPS.

### 2.9. MTT Cell Viability Assay

The cells were plated at a density of 1 × 10^5^ cells per well in a 24-well plate to determine any potential cytotoxicity [12]. Cells were serum starved for 12 h and then treated with EER for the next 24 h. After incubation of the cells, viable cells were stained with MTT (0.5 *μ*g/mL, 4 h). The media were then removed, and the produced formazan crystals in the wells were dissolved by addition of 300 *μ*L dimethyl sulfoxide. Absorbance was measured at 570 nm using a Titertek Multiskan Automatic ELISA microplate reader (Model MCC/340, Huntsville, AL, USA). Cell viability was defined relative to untreated control cells (i.e., viability (% control) = 100 × (absorbance of treated sample)/(absorbance of control)).

### 2.10. Assay of Nitrite Production

NO production was monitored by measuring the nitrite content in culture medium [12]. This was performed by mixing the samples with Griess reagent (1% sulfanilamide, 0.1% N-1-naphthylenediamine dihydrochloride, and 2.5% phosphoric acid). Absorbance was measured at 540 nm after incubation for 10 min.

### 2.11. Enzyme-Linked Immunosorbent Assay (ELISA)

Raw264.7 cells were preincubated with EER for 1 h and continuously incubated with LPS for 24 h [12]. TNF-*α*, IL-1*β* (Endogen, Woburn, MA, USA), and PGE_2_ (RnD Systems, Minneapolis, MN, USA) contents in the culture medium were measured by ELISA using antimouse TNF-*α*, IL-1*β*, and PGE_2_ antibodies and biotinylated secondary antibody according to the manufacturer's instruction.

### 2.12. Immunoblot Analysis

Cells were lysed in the buffer containing 20 mm Tris·HCl (pH 7.5), 1% Triton X-100, 137 mm sodium chloride, 10% glycerol, 2 mm EDTA, 1 mm sodium orthovanadate, 25 mm b-glycerophosphate, 2 mm sodium pyrophosphate, 1 mm phenyl methyl sulfonyl fluoride, and 1 mg/mL leupeptin [12]. Cell lysates were centrifuged at 15,000 g for 10 min to remove debris. The immunoreactive bands were visualized using enhanced chemiluminescence (ECL) detection kit (Amersham Biosciences Corp., Piscataway, NJ, USA) according to the manufacturer's instructions. Eual loading of proteins was verified by actin or lamin immunoblottings. 

### 2.13. Scanning Densitometry

Densitometric measurements of the bands were made using an image analyzing system (Ultra-Violet Products Ltd., Upland, CA, USA). Repeated experiments were separately performed to confirm changes.

### 2.14. Statistical Analysis

One-way analysis of variance (ANOVA) was used to assess statistical significance of differences among treatment groups. For each statistically significant effect of treatment, the Newman-Keuls test was used for comparisons between multiple group means. The data were expressed as means ± 95% confidence intervals (CI). All statistical tests were two sided.

## 3. Results

### 3.1. Inhibition of Carrageenan-Induced Paw Edema

**Figure 1:**
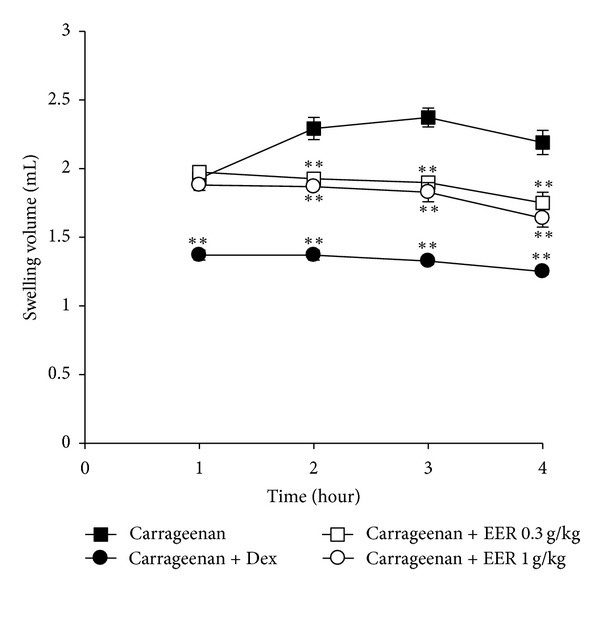
Inhibition of carrageenan-induced paw edema formation by EER. The EER was administered to rats at the oral dose of 0.3 or 1 g/kg/day. Then, paw edema was induced by subcutaneously injecting 1% solution of carrageenan dissolved in saline (0.1 mL per animal) into the hind paw. The swelling volume of the paw was measured at 1–4 h after carrageenan injection. Dexamethasone (Dex, 1 mg/kg, p.o.) was used as a positive control. Data represents the mean ±  S.E.M. of five animals (significant as compared with carrageenan alone, **P < 0.01). For data points where error bars could not be seen, the standard error was subtended by the data point. EERs: extract of *Erigeron annuus* roots.

To verify inhibitory effects of EER on acute inflammation *in vivo*, we used the carrageenan-induced paw edema model, which is a well-established model for screening the efficacy of anti-inflammatory drugs [24]. Paw edema formation by carrageenan was observed from 1 h and persisted at least up to 4 h after injection (Figure 1). Maximal induction of paw swelling by carrageenan was observed at 3 h. Administration of EER (0.3 and 1 g/kg/day, p.o., for 4 days) inhibited the ability of carrageenan to induce paw swelling. We also confirmed that dexamethasone (1 mg/kg/day, p.o., for 4 days), a positive control, inhibits the edema formation. 

### 3.2. Inhibition of Carrageenan-Induced Acute Inflammation

**Figure 2:**
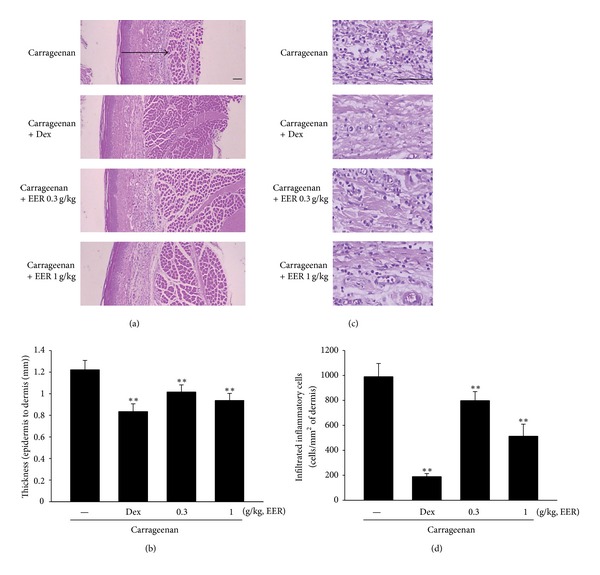
Inhibition of carrageenan-induced paw swelling and infiltration of inflammatory cells by EER. (a) Histomorphometry of the *ventrum pedis* skin. In each rats, cutaneous regions of *ventrum pedis* were stained with H and E and used for histological sample preparation in this study. Arrow indicated total thicknesses measured. Scale bars = 160 *μ*m. (b) Thickness of epidermis to dermis. Data represents the mean ± S.E.M. of five animals (significant as compared with carrageenan alone,  **P < 0.01). (c) Histopathology of the *ventrum pedis* stained with H and E. Scale bars = 160 *μ*m. (d) Infiltration of inflammatory cells. Data represents the mean ± S.E.M. of five animals (significant as compared with carrageenan alone, **P < 0.01). EERs: extract of *Erigeron annuus* roots.

In addition, we confirmed the effects of EER on histological profiles of *ventrum pedis* skin stained with H and E. As shown in Figure 2(a), carrageenan successfully induced paw swelling in *ventrum pedis* in rats. However, pretreatment of EER at the oral doses of 0.3 and 1 g/kg for 4 days significantly blocked the changes of the thickness of *ventrum pedis *(Figure 2(b)). Moreover, injection of carrageenan markedly increased infiltration of inflammatory cells, which were inhibited by treatment of EER (Figures 2(c) and 2(d)). Dexamethasone also decreased the number of infiltrated inflammatory cells. These results suggest that repression of paw swelling and of infiltration of inflammatory cells by EER may represent an important efficacy in association with the inhibition of acute inflammation in rats. 

### 3.3. Inhibition of LPS-Inducible NO, PGE_2_, and Proinflammatory Cytokines Production

**Figure 3:**
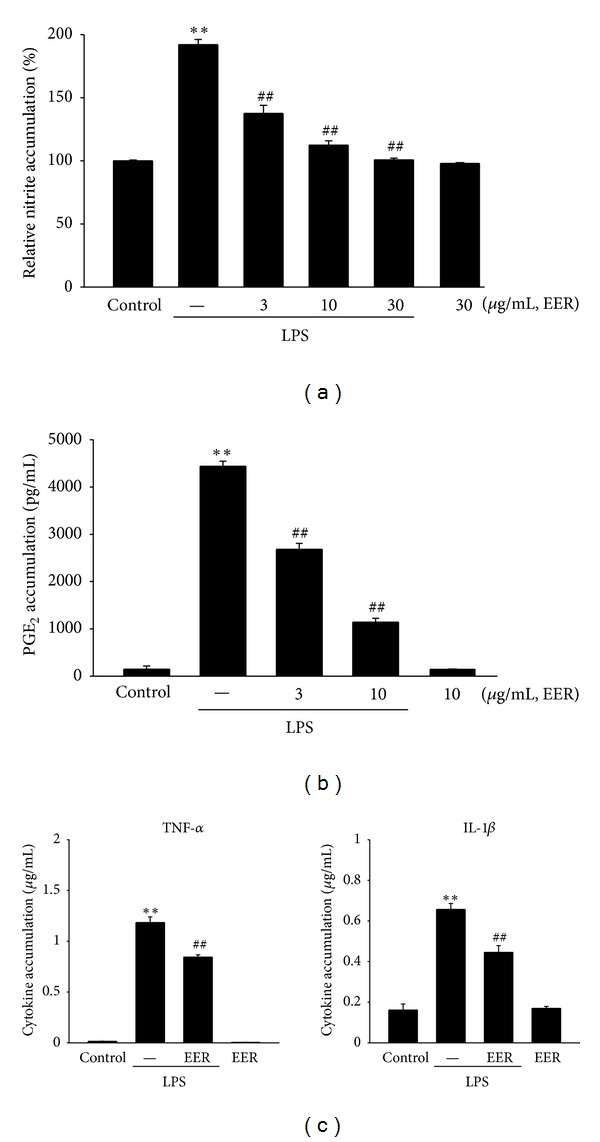
Inhibition of LPS-inducible NO and PGE_2_ by EER. (a) NO and (b) PGE_2_ accumulations. Raw264.7 cells were treated with EER at the indicated doses for 1 h and continuously incubated with LPS (1 *μ*g/mL) for the next 24 h. (c) TNF-*α* and IL-1*β* contents in culture medium. Raw264.7 cells were stimulated by LPS with or without 10 *μ*g/mL EER for 24 h. Data represents the mean ± S.E.M. from four separate experiments (significant as compared with vehicle-treated control, **P < 0.01; significant as compared with LPS alone, ^##^ *P* < 0.01). EERs: extract of *Erigeron annuus* roots.

Next, our studies were extended to determine the effects of EER *in vitro*. First, we examined any possible toxicity of EER in Raw264.7 cells. In MTT assay, cell viability was not affected by EER treatment up to 30 *μ*g/mL (data not shown). We chose 3–30 *μ*g/mL concentrations of EER to determine the effect of EER on NO accumulation in LPS-stimulated Raw264.7 cells. LPS (1 *μ*g/mL, 24 h) significantly increased NO production, which was markedly inhibited by EER (3, 10, and 30 *μ*g/mL) in a concentration-dependent manner (i.e., 29%–50%) (Figure 3(a)). PGE_2_ production in LPS-treated Raw264.7 cells was also inhibited by EER (3 and 10 *μ*g/mL) (Figure 3(b)).

Cytokine is known as one of the major mediators in inflammatory responses. To confirm the inhibition of proinflammatory cytokines, we verified the effects of EER on TNF-*α* and IL-1*β* productions in Raw264.7 cells. LPS stimulation significantly increased the productions of TNF-*α* and IL-1*β* in culture media of Raw264.7 cells (Figure 3(c)). Treatment of EER at 10 *μ*g/mL inhibited the ability of LPS to induce the cytokines accumulation (Figure 3(c)). Moreover, EER treatment alone had no effects on macrophage activation. These results suggest that EER blocked the activation of macrophage cells in terms of inhibition of NO, PGE_2_, and cytokines. 

### 3.4. Inhibition of LPS-Inducible iNOS and COX-2 Protein Expression

**Figure 4:**
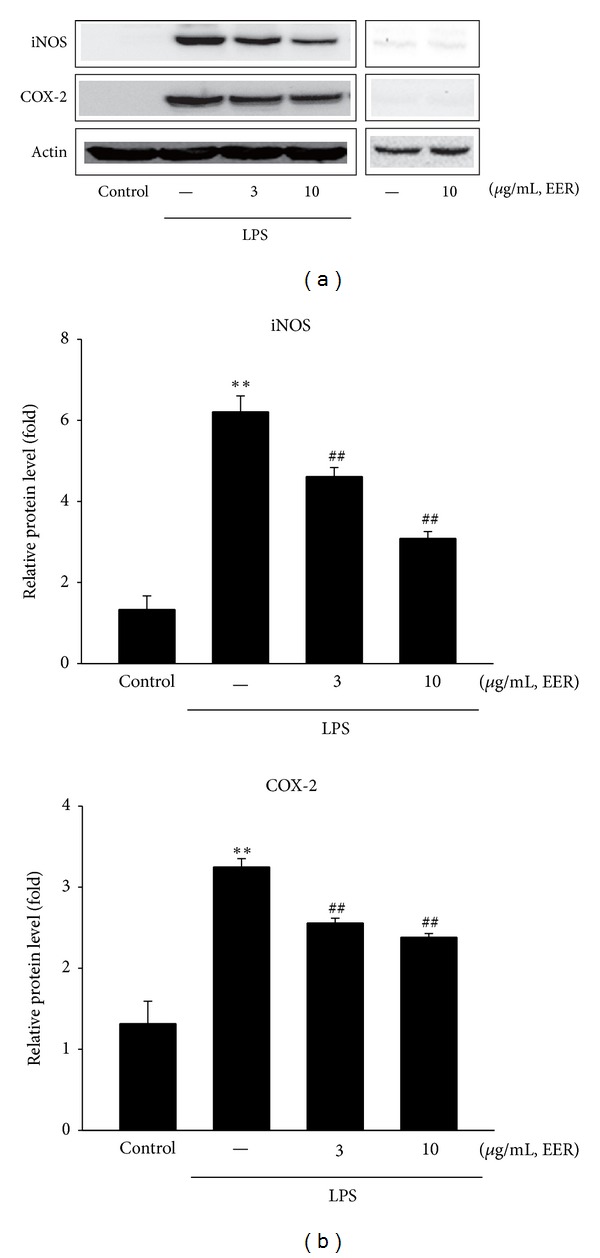
Inhibition of LPS-inducible iNOS and COX-2 by EER. (a) Representative iNOS and COX-2 immunoblottings. iNOS or COX-2 protein levels were monitored 18 h after treatment with LPS. (b) Relative iNOS and COX-2 protein levels. Data represents the mean ± S.E.M. from three separate experiments (significant as compared with vehicle-treated control, **P < 0.01; significant as compared with LPS alone, ^##^ *P* < 0.01). EERs: extract of *Erigeron annuus* roots.

iNOS and COX-2 are key enzymes in the pathophysiological process of inflammation and the induction of inflammatory mediators such as NO and PGE_2_. Next, we determined the protein expressions of iNOS and COX-2 by western blot analysis. LPS treatment at 1 *μ*g/mL for 18 h significantly induced protein levels of iNOS and COX-2 in Raw264.7 cells (Figure 4(a)). Densitometer analysis revealed that EER treatment (3 and 10 *μ*g/mL) at 1 h prior to LPS significantly inhibited the iNOS and COX-2 protein expressions in Raw264.7 cells (Figure 4(b)).

### 3.5. Inhibition of LPS-Inducible NF-*κ*B Activation

**Figure 5:**
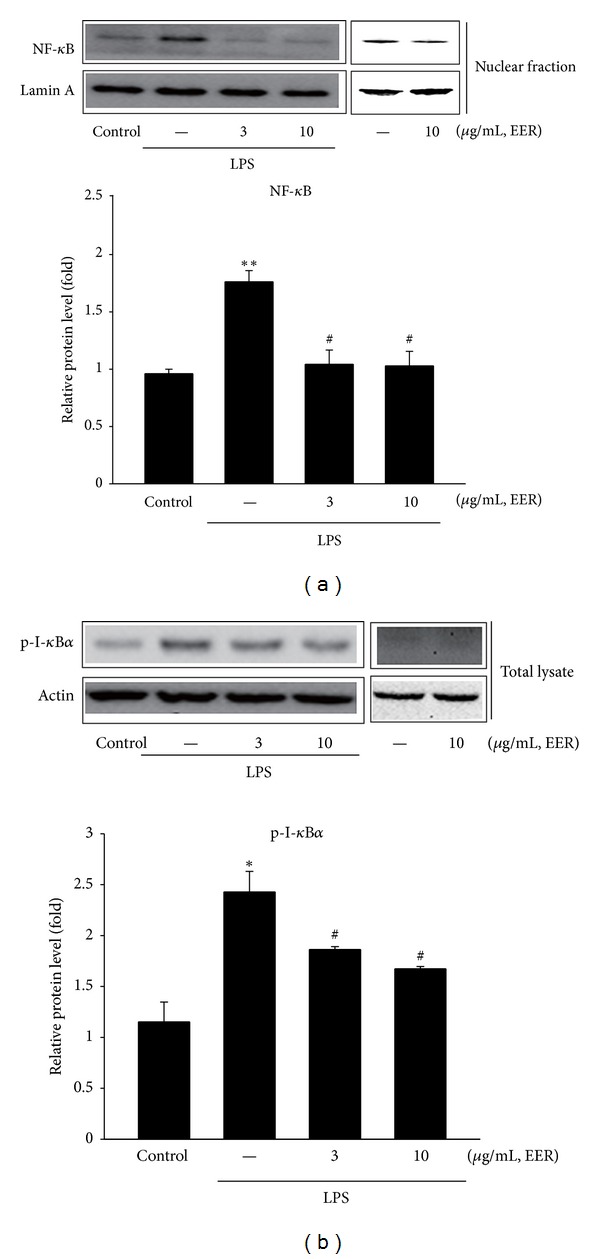
Inhibition of LPS-induced NF-*κ*B activation by EER. (a) The level of NF-*κ*B protein in nucleus. The cells were treated with LPS or LPS + EER for 1 h. Immunoblottings for lamin A verified equal loading and purity of the nuclear proteins. (b) The level of phosphorylated I-*κ*B*α* (p-I-*κ*B*α*) in total lysates. Raw264.7 cells were treated with LPS or LPS + EER for 30 min. Immunoblots are representative results from repeated experiments. Data represents the mean ± S.E.M. from three separate experiments (significant as compared with vehicle-treated control,  *P < 0.05, **P < 0.01; significant as compared with LPS alone, ^#^ *P* < 0.05). EERs: extract of *Erigeron annuus* roots.

NF-*κ*B is one of the most important transcription factors for the induction of the inflammatory genes such as iNOS and COX-2 in immune cells stimulated with inflammatory inducers including LPS [17–19]. NF-*κ*B is translocated to the nucleus by phosphorylation of I-*κ*B*α* and subsequent degradation of I-*κ*B*α* subunit. Treatment of Raw264.7 cells with EER for 1 h markedly inhibited the nuclear level of NF-*κ*B induced by LPS (Figure 5(a)). Moreover, exposure of LPS decreased the increased phosphorylation of I-*κ*B*α* protein level at 30 min, which was also blocked subsequent treatment of EER (Figure 5(b)). These results suggest that EER might prevent translocation of NF-*κ*B to the nucleus by inhibiting I-*κ*B*α* phosphorylation.

## 4. Discussion

*E. annuus* is called the annual fleabane, which is a plant indigenous to Eastern North America widely found at meadows and roadside [20]. It is well known to be used as the treatment of indigestion, epidemic hepatitis, enteritis, lymphadenitis, and hematuria in traditional oriental medicine [25, 26]. Recently, it has been studied that each part of *E. annuus* has different pharmaceutical efficacies. The flower extract *E. annuus* had anti-inflammatory effect through the induction of hemeoxygenase-1, and its leaf extract was shown to have neuroprotective and antioxidant effects against oxidative stress induced by H_2_O_2_ [27, 28]. Nevertheless, the scientific proof and mechanistic basis for the effect of *E. annuus* roots have almost not been studied. In this study, we determined anti-inflammatory effects of EER *in vivo* and *in vitro* for the first time. 

Swelling is one of the most important symptoms of acute inflammation and characterized by an increase in vascular permeability and infiltration of cells. Therefore, its induction by carrageenan is a well-established model to screen novel anti-inflammatory drug [22, 29, 30]. In our study, carrageenan injection successfully creates acute inflammation in the peripheral tissue, as indicated by increases in the number of inflammatory cells and skin thicknesses in *ventrum pedis*. Also, carrageenan induced paw swelling during 1–4 h (maximally 3 h) after injection. These results are consistent with previous observations, showing that carrageenan induces infiltration of immune cells and resultantly increased inflammatory response [29, 30]. Next, we assessed the effects of EER in rats injected with carrageenan. Administration of EER inhibited the infiltration of inflammatory cell in the tissue and swelling in the paw skin. These results, here, demonstrate that EER could prevent the acute inflammation *in vivo.*

The pathology of inflammation is started by complex processes stimulated by the endotoxins such as LPS. In this immune defense mechanism, macrophages play an important role in the progression of the human disorder [8]. LPS can directly activate macrophages, which produce the inflammatory mediators such as NO, eicosanoids, TNF-*α*, and ILs [9, 10]. TNF-*α* and IL-1*β*, the representative proinflammatory cytokines, are small secreted proteins mediating immunity and inflammation. TNF-*α* can activate macrophages and initiate immune responses by stimulating secretion of other cytokines. IL-1*β*, mainly synthesized by macrophages, is also inflammatory cytokines which play important roles in the acute phase response.

Here, we evaluated the effects of EER on the accumulations of NO and PGE_2_ in media of Raw264.7 cells stimulated by LPS. Pretreatment of EER suppressed production of LPS-inducible NO and  PGE_2_.  In addition, EER treatment significantly blocked the production of proinflammatory cytokines, TNF-*α*, and IL-1*β*. Moreover, it has been shown that carrageenan promotes the release of NO as well as PGE_2_ in the peripheral tissue [31]. Chuha et al. have also shown that carrageenan stimulates the release of TNF-*α* and ILs. In view of the inhibitory effects of EER on NO and cytokines productions *in vitro* and the important role of NO and PGE_2_ on swelling *in vivo*, the effects of EER on acute inflammation in rats might result, at least in part, from its inhibition of NO and PGE_2_ in the tissues.

NO and PGs play key roles in defense mechanisms against xenobiotic stimuli such as infection; it has been perceived that the proteins (i.e., NOS or COX) are main regulators having harmful role in the pathological process of inflammation. Coffey et al. showed that macrophages from iNOS knock-out animals are protected tissue from acute inflammation [32]. In case of eicosanoids, it has been shown that production of a large amount of PGs have detrimental effects in inflammation-related diseases, which were mainly dependent on the COX. Immunoblot analysis enables us to verify that the treatment of EER significantly suppressed the ability of LPS to induce iNOS and COX-2. 

NF-*κ*B is one of important transcription factors in terms of regulation of the genes, which were involved in inflammatory and immune responses, cell adhesion, and survival [17, 18, 33]. Degradation of I-*κ*B*α* by its phosphorylation causes activation of NF-*κ*B, which enables NF-*κ*B itself to translocate into the nucleus to initiate transcription of the target genes. In this study, we assessed immunoblot analysis to show the effects of EER on NF-*κ*B activation. As shown in Figure 5, pretreatment of EER inhibited phosphorylation of I-*κ*B*α* induced by LPS and resultant degradation of I-*κ*B*α* (data not shown). Also, EER treatment markedly blocked the nuclear level of NF-*κ*B, showing that the effect of EER on the expression of inflammatory genes might result from the inhibition of NF-*κ*B activation. Moreover, it has been shown that I-*κ*B*α* is phosphorylated by I-*κ*B*α*  kinase (IKK) complex, which may be activated by a line of kinases (e.g., tyrosine kinase family members and mitogen-activated protein kinases) [34, 35]. It might have value to study the effects of EER on these upstream kinases, which may have the possibility of being a potential pharmacological target of EER.

The chemical compositions of *E. annuus* have been reported by many researchers. Flower of *E. annuus *has a large number of phenolic compounds [36]. The three quinic acid derivatives and four flavonoids were isolated from the leaves and stems of *E. annuus* [25]. The aerial parts or the roots of *E. annuus* contained sesquiterpenoids, diterpenoid, polyacetylenic compound, *γ*-pyrone derivatives, sterols, triterpenoids, monoterpene hydrocarbons, and monoterpene oxygenated components [37, 38]. Among them, it has been shown that sesquiterpenoids, diterpenoid, sterols, and triterpenoids had anti-inflammatory effects [39–46]. Therefore, it remains to be confirmed what is pharmacologically effective ingredient in the EER in terms of anti-inflammation.

## 5. Conclusions

In our study, we used two approaches to show the effect of EER: (1) an animal model using carrageenan-induced acute inflammation and (2) a cell model using activated macrophage induced by LPS. Oral administrations of EER attenuated acute inflammation, as indicated by the inhibition of inflammatory cells infiltration and paw swelling induced by carrageenan. Treatment of EER in Raw264.7 cells suppressed the activation of the macrophage by preventing the induction of iNOS and COX-2 protein expressions as well as the productions of NO and PGE_2_, which is related with its inhibition of NF-*κ*B activation. These findings showing here might help to understand the pharmacology of the roots of* E. annuus* and offer the possibility of herbal candidate for the treatment of inflammatory disease. 

## Supplementary Material

Analysis of EER was performed on an Agilent 6890 gas chromatography equipped with a 5975 GC/MS selective detector (agilent, CA, USA). Mass spectral analyses were performed using the NIST05 library resident in the computer. Percentage composition was calculated using the area normalization method.

## Abbreviations

COX: Cyclooxygenase
EER: Extract of *Erigeron annuus* root
I-*κ*B: Inhibitor of *κ*B
IL: Interleukin
iNOS: Inducible nitric oxide synthase
LPS: lipopolysaccharide
NF-*κ*B: Nuclear factor-*κ*B
NO: Nitric oxide
PGE: Prostaglandin E
TNF-*α*: Tumor necrosis factor-*α*.
