# Supplementary material for: Roots of Erigeron annuus Attenuate Acute Inflammation as Mediated with the Inhibition of NF-κB-Associated Nitric Oxide and Prostaglandin E2 production
Source: Evid Based Complement Alternat Med. 2013 Feb 24;2013:297427. doi: 10.1155/2013/297427 (PMC3595701; doi:10.1155/2013/297427)
Supplement: Supplementary file 1 — Analysis of EER was performed on an Agilent 6890 gas chromatography equipped with a 5975 GC/MS selective detector (agilent, CA, USA). Mass spectral analyses were performed using the NIST05 library resident in the computer. Percentage composition was calculated using the area normalization method. [file 297427.f1.doc]

**Supplementary Figure legend**

Supple. Figure S1. Analysis of Extract of *Erigeron annuus* Roots (EER) by gas chromatography and mass spectrometry. The gas chromatogram of EER with peaks at retention times (a) 30.120 (3,4-Ethylenedisulfanylthiophene), (b) 39.317 (Methyl hexadecanoate), (c) 40.060 (Palmitinic acid) and (d) 43.051 (Methyl linoleate).
